# The Physicians’ Visit to the Islands

**Published:** 1853-06

**Authors:** 


The following day an excursion was made to the Public Charities in the neighborhood of the city, for a description of which we are again indebted to a New York paper:

THE PHYSICIANS' VISIT TO THE ISLANDS.

Yesterday the medical guests of the City Physicians' surrendered themseves into the hands of the Committee, headed by Dr. Griscom. According to arrangement they met—a goodly number of them—at Pier No. 3, North River, on board the steamer Hero, at 9 A. M., and were speedily under way. As to the weather, it was the best the day afforded—cloudy, but that prevented the glare of the sun on the water, which is so annoying to gentlemen from the interior,—cold, but that obviated the unpleasant heat of the first of the week,—rainy, but nearly half of them had umbrellas,—muddy under foot, but at least a dozen pair of rubbers were in the company. Having cruised about the harbor from Weehawken to Fort Hamilton for a couple of hours, the little steamer called at the foot of Grand street, took on an additional number,—mostly of those who had made their morning calls, prescribed the pills and potions, and pocketed the fees of the day,—and off they started for the islands. They called at Ward's Island and under the escort of Dr. Cox noticed the various forms of disease abundantly presented in the Emigrants' Hospital.

They stopped longer at Randall's Island, where the simple songs of the children, the exercises of the school, and the thought of the utter loneliness which once in a while must break in upon the young hearts of these little outcasts, deeply affected their visitors. Dr. Worthington Hooker and Dr. Charles Hooker, both addressed the little ones in an appropriate strain.

Here it was found necessary to levy liberally upon the sandwiches and their collaterals, to satisfy the cravings of the inner man. On board again, it was deemed wise to dip pretty largely into the pickled oysters and eat heartily of the cold turkey, for fear of a lack of strength to endure the dinner in prospect.

Landed at Blackwell's Island, some took to the Penitentiary, some to the Lunatic Asylum, some visited the Work-houses, and some hurried through the Hospital-wards with Dr. Kelly, catching rapt glances of beautiful specimens of ulcers and of physical deformity; a seemingly infinite variety. Each one wandered according to his taste. It would have done you good to have seen them wading through the mud in prosecution of said wanderings. Men from the country as they forded it talked naturally of subsoiling, and citizens consoled themselves with thinking how the walking must be in Broadway, if it was so bad in the green and beautiful country. How the ladies—quite a large number of them accompanied the party, and by their presence made the darkness of the sky tolerably light, and the mud on their boots less intolerably weighty—how the ladies managed to get through at all was a marvel. They all did get through, however, in time to grace the tables which reached the whole length of the new Workhouse Hall, when 4 o'clock, the hour for dinner, arrived. The dinner was good, and things took such a turn that speech-making came "mighty easy" in its season. Stirring addresses, principally regarding the ethics of the profession, were made by Dr. F. Campbell Stewart, Dr. Stone, Dr. James Wood, Dr. Atlee, Dr. Griscom, and Mr. West, the President of the Board of Governors, who presided. With three cheers for the Governors, whose guests the company were, and three for the Committee of Arrangements, who planned so excellently every thing but the weather, an adjournment was taken. Escorted by the boys in excellent style and time the company took to the boat. A meeting was here organized, Dr. Worthington Hooker called to the chair, and Dr. Gooch made Secretary. Resolutions of thanks to the Ten Governors and to the Commiitee of Arrangements, were adopted, and, at 6½ o'clock, the landing at the foot of Grand street was effected.

The hospitality of the physicians and citizens of New York did not stop with the superb banquet at Metropolitan Hall, and the festivities attendant upon this pleasant excursion. Houses were open to the members of the Association every evening, and New York seemed resolved to show that warm hearts were not confined to the South. On successive evenings the Association was entertained by Ex-Governor Fish, Ex-Mayor Kingsland, Prof. Parker, Dr. Isaac Wood, Dr. James R. Wood, Dr. Cammann, Dr. Cheeseman, Dr, John Watson, Prof. Green, Dr. Lewis Sayre, Dr. Detmold, and other eminent physicians.

The Committee of Arrangements discharged their duty admirably. Everything was tastefully and judiciously arranged, and the impression left upon the minds of all the members must have been, that if any future meeting of the Association ever equals, it is impossible that one can ever surpass in interest the meeting at New York.

We shall look with impatience for the next volume of transactions, which will embrace some of the best papers ever called forth by the Association.

In reviewing these proceedings the reader will not fail to observe, that legislation formed but a small part of the business of the meeting, and that all the radical changes in the constitution proposed a year ago were indefinitely postponed. There seemed, indeed, not much disposition to discuss these amendments, or to enter the wide field of medical politics. The members had met to recieve scientific reports, to advance medical science, to interchange friendly greetings, to cement the bonds of professional brotherhood, and to enjoy New-York. For ourselves, we have to express our perfect and entire satisfaction with the result; we do not believe the meeting could have been occupied with worthier objects. But not so does it appear to many of our brethren, who, if they cannot have legislation, are not willing to accept of anything. The amendments of the constitution, in their view, were the great objects before the meeting, and these having failed, all has failed. To their minds, the Association is a potent law-making body, and its vocation is, especially, to regulate the medical schools of the country. The scientific reports of committees, the prize essays, the social influences of the Association, its liberalizing, elevating tendencies, all pass with them for nothing so long as it neglects to prescribe a course of conduct for the schools. To them, the profession seems to be running down, and nothing but a regulation of the schools by the Association can save it.

We were gratified to perceive, as we think we did, that these views are not as prevalent as they were at some of the earlier meetings of the Association. They have always struck us as unjust and visionary. The evils complained of have but slight foundation in fact, and even if real the remedies proposed for them are impracticable. We believe, with the amiable, learned, and accomplished President of the Association, that the profession "has steadily advanced." It was never so skilful, devoted, and efficient as now, and never enjoyed more of the public esteem. The Association will impel it forward in the right course, not by regulating the medical schools, but by infusing into it a higher tone, and stimulating the pride and industry of its members.

